# A National Overview of Nutritional Care in Diet-Treated Inborn Errors of Metabolism in Brazil

**DOI:** 10.3390/ijerph23060807

**Published:** 2026-06-17

**Authors:** Soraia Poloni, Laura de Azevedo Pesce, Viviane de Cássia Kanufre, Lilia Ramos Farret, Camila Pugliese, José Araújo de Oliveira Silva, Monique Poubel, Maria Efigênia de Queiroz Leite, Renata Bernardes de Oliveira, on behalf of the SBTEIM Nutrition Department

**Affiliations:** 1 Hospital de Clínicas de Porto Alegre, Rua Ramiro Barcelos, 2350, Porto Alegre 90035-903, Brazil; lapesce@hcpa.edu.br (L.d.A.P.); lrefosco@hcpa.edu.br (L.R.F.); 2 Hospital das Clínicas da UFMG/Ebserh, Núcleo de Ações e Pesquisa em Apoio Diagnóstico, NUPAD-UFMG, Belo Horizonte 30130-100, Brazil; vikanufre@gmail.com; 3 Instituto da Criança e do Adolescente do Hospital das Clínicas da Faculdade de Medicina da Universidade de São Paulo, São Paulo 05403-010, Brazil; camila.pugliese@hc.fm.usp.br; 4 Instituto de Genética e Erros Inatos do Metabolismo IGEIM/CREIM/UNIFESP, São Paulo 04020-041, Brazil; jose.araujo@unifesp.br (J.A.d.O.S.); oliveira.b.renata@gmail.com (R.B.d.O.); 5 Centro de Referência de Doenças Raras e Serviço de Referência em Triagem Neonatal do Distrito Federal HAB/SES-DF, Hospital de Apoio de Brasília, Brasília 70684-831, Brazil; monolivepoubel@gmail.com; 6 Serviço de Referência em Triagem Neonatal APAE, Salvador 41830-141, Brazil; maria-leite.ml@ebserh.gov.br

**Keywords:** inborn errors of metabolism, dietary management, phenylketonuria, aminoacidopathies, glycogen storage disease

## Abstract

**Highlights:**

**Public health relevance—How does this work relate to a public health issue?**

- Dietary therapy is a life-sustaining intervention for many IEM, yet this study demonstrates substantial gaps in access to metabolic formulas, special low-protein foods, and trained professionals within the Brazilian public health system, directly impacting disease control, growth, and neurodevelopment outcomes.
- By highlighting deficiencies in professional training, resource availability, and socioeconomic barriers (including food insecurity), this work provides actionable evidence to guide public health policies aimed at strengthening specialized care networks, improving equity, and optimizing long-term outcomes for individuals with rare metabolic disorders.

**Public health significance—Why is this work of significance to public health?**

- By revealing gaps in access, adherence, and support, this study highlights missed opportunities to reduce preventable disease burden and lifelong disability.
- The findings expose disparities in resources, training, and food security that compromise care delivery. Addressing these gaps can improve health equity, optimize the use of public resources, and reduce downstream healthcare costs associated with poorly controlled metabolic disorders.

**Public health implications—What are the key implications or messages for practitioners, policymakers, and/or researchers in public health?**

- This study underscores the need to generate evidence that supports strengthening national policies to ensure equitable access to metabolic formulas and special low-protein foods, the integration of IEM care into structured rare disease networks, and investment in workforce training to reduce regional disparities and improve continuity of care.
- For practitioners and health services, implementing standardized dietary management protocols, strengthening multidisciplinary care models, and incorporating patient education strategies that address health literacy and food insecurity can improve adherence and clinical outcomes.

**Abstract:**

Aim: To evaluate the status of the nutritional management of diet-treated IEM in Brazil from the perspectives of healthcare professionals, patients, and families. Methods: Data were collected through two nationwide digital questionnaires administered to healthcare professionals involved in dietary management (*n* = 37) and to patients and caregivers (*n* = 278), addressing professional training, workload, access to resources, treatment adherence, and socioeconomic factors. Results: Healthcare professionals from 20 out of the 26 Brazilian states participated, most of them female (81%) and dietitians (81%). Although more than half had over 10 years of experience, 59% considered their training insufficient to work with IEM. Only 19% reported exclusive dedication to the field, and 54% were the sole professional responsible for dietary prescriptions at their center. Weekly workload dedicated to IEM varied widely. Among the patients and families, phenylketonuria (60.4%) and glycogen storage disease (25.9%) were the most frequent conditions. Higher educational level and longer time since diagnosis were associated with a better understanding of dietary management (*p* < 0.05). Among patients on protein-restricted diets, most reported regular use of protein substitutes, although 92% reported poor palatability and 36% reported supply problems. Access to special low-protein foods was limited, and over half of the families reported some level of food insecurity. Conclusions: Significant systemic, logistical, and socioeconomic barriers to optimal dietary management of IEM persist in Brazil, highlighting the need for strengthened public policies, professional training, and equitable access to dietary resources.

## 1. Introduction

For many inborn errors of metabolism (IEM), dietary management represents the cornerstone of disease-modifying treatment, functioning as a precision therapy that modulates the intake of specific dietary substrates to maintain metabolic homeostasis [1,2,3]. This strict dietary management, which often involves the use of specialized nutritional products, is a key pillar for toxicity control and for ensuring adequate growth and neuropsychomotor development in affected patients. However, the effectiveness of this intervention intrinsically depends on its continuous and specialized implementation, as well as on unrestricted access to dietetic supplies [1,4].

The progressive and often irreversible nature of sequelae associated with IEM underscores the critical importance of early diagnosis and prompt therapeutic intervention. In this context, newborn screening emerges as a high-impact public health strategy, enabling presymptomatic identification of several IEM. In Brazil, the National Newborn Screening Program, managed by the Unified Health System (Sistema Único de Saúde—SUS), has included screening for phenylketonuria, congenital hypothyroidism, sickle cell disease and cystic fibrosis since 2001 and is currently undergoing expansion to encompass more than 50 conditions (aminoacidopathies, organic acidurias, lysosomal storage diseases, primary immunodeficiencies, and spinal muscular atrophy), most of which are primarily managed through dietary treatment [5]. Currently, the country has 29 reference centers for newborn screening, which provide follow-up care for patients screened through SUS.

Despite advances in screening and the establishment of clinical guidelines, the logistical complexity and regional diversity of Brazil pose considerable challenges to the uniformity and quality of nutritional care provided by the SUS [6]. Furthermore, although this is a highly specialized area within nutrition practice, there are no specific professional practice guidelines or formal recognition of specialists in this field in Brazil.

Therefore, the aim of this study was to assess the current status of nutritional management of IEM primarily treated with dietary interventions within the SUS across different regions of Brazil, considering both the experience of healthcare professionals working in this field and the perspectives of patients and family members living with these conditions.

## 2. Materials and Methods

Two digital questionnaires were developed by a team of expert Brazilian metabolic dietitians and implemented through the Zoho Forms platform (Zoho Corporation Pvt. Ltd., Chennai, Tamil Nadu, India). Questionnaire 1 was directed at healthcare professionals, whereas Questionnaire 2 targeted patients and family members living with an IEM under dietary treatment. Both questionnaires included items related to the nutritional management of IEM, with a particular focus on disorders requiring protein-restricted diets—especially phenylketonuria—and hepatic glycogen storage diseases. These conditions were selected because they are relatively prevalent in the country, have well-established dietary treatments, and require the daily and regular use of special foods as part of therapy. Prior to data collection, the questionnaires were pilot-tested and validated with 10 individuals, including healthcare professionals and patients, to assess clarity, relevance, and comprehensibility of the items.

Questionnaire 1 had 40 questions and was addressed to healthcare professionals who directly prescribe dietary treatment (dietitians or physicians) and work in follow-up and treatment services for patients with IEM within the Brazilian Unified Health System (*SUS*). The questionnaire comprised multiple-choice and short-answer questions and explored professional training, clinical practices, available resources, and barriers to providing adequate dietary care. Healthcare professionals were contacted through Brazilian genetics societies and reference centers via email invitations containing the access link and detailed information about the study.

Questionnaire 2 was intended for patients aged 18 years or older, or for family members of patients of any age, diagnosed with an IEM under dietary treatment and followed at a treatment center in Brazil. It focused on access to dietary products, understanding of the prescribed diet, adherence, and the impact of dietary therapy on daily life. The validated short screening version (TRIA) of the Brazilian Food Insecurity Scale (EBIA), consisting of two questions, was used to identify the risk of food insecurity in this population [7]. Recruitment and study invitations were carried out through nationwide patient associations (Mães Metabólicas and AbGlico), as well as through dissemination of the study link and invitation on the Instagram social media platform via the @nutricaometabolica account, which is aimed at professionals working in the fields of nutrition and metabolism.

### Ethical Aspects

The study was approved by the Research Ethics Committee of the Hospital de Clínicas de Porto Alegre, Brazil (CAAE 74930323.5.0000.5327). Participation was confirmed by the submission of the completed questionnaire, after agreement with the informed consent terms presented prior to the start of the survey.

## 3. Results

### 3.1. Healthcare Professionals

Responses were obtained from 37 healthcare professionals across 20 out of the 26 Brazilian states. The characteristics of this sample are presented in Table 1.

More than half of the respondents (51%) had over 10 years of professional experience; however, 59% considered their education and/or training insufficient to work in the field of IEM. Only seven professionals (19%) reported exclusive dedication to IEM, and 20 (54%) stated that they were the sole professionals responsible for dietary prescription at their treatment centers. Twenty-eight respondents (76%) provided care for patients with phenylketonuria (PKU) in conjunction with other IEM, while five (14%) reported working exclusively with PKU.

Regarding resources for dietary management, most professionals (*n* = 30; 81%) did not have access to dietary calculation software, relying instead on manual calculations (*n* = 17), self-developed spreadsheets (*n* = 10), or simplified prescription methods (*n* = 3). Concerning the availability of laboratory tests essential for dietary management, 21 out of 37 respondents (56%) reported adequate availability, whereas 13 out of 37 (35%) reported availability below what is required, and 3 out of 37 (8.1%) reported complete unavailability of these tests. Only 6 out of 37 respondents (16%) reported the existence of a local (municipal or state-level) program for the provision of special low-protein foods. When asked about the supply of metabolic formulas (designed for inborn errors of protein metabolism) over the previous 12 months, only 24% of respondents reported regular provision by the state, while 40% indicated inconsistencies in supply affecting all disorders requiring these products, with disruptions impacting more than 10 patients at their treatment centers. In Brazil, access to these products through the Federal or State Government frequently depends on legal action.

### 3.2. Patient Experience Results

A total of 278 responses were obtained, of which 23 (8.3%) were completed by the patients themselves, 220 (79.2%) by mothers, and the remaining 35 (12.5%) by other family members. Regarding geographic origin, most families were from the Southeast region of Brazil (*n* = 136; 48.9%), followed by the South (*n* = 64; 23.0%), Northeast (*n* = 38; 13.7%), Midwest (*n* = 30; 10.8%), and North (*n* = 10; 3.6%). Additional characteristics of the evaluated sample are presented in Table 2. The educational level presented here refers to the individual who completed the questionnaire.

In 59% of cases, the diagnosis had been established for ≥5 years, and 59.7% of respondents reported that the diagnosis was made through newborn screening (all of them with PKU), followed by 38.8% of cases diagnosed after the onset of symptoms (14% with PKU and the remaining with other IEM).

Dietitians were identified as the primary healthcare professionals responsible for dietary management in 88.1% of cases and were also the main resource used by families for clarification of diet-related questions (59.7%), followed by counseling from other families with the same diagnosis (26.6%). Regarding self-perceived metabolic control, 73% of respondents considered their metabolic control to be good or excellent, 22.7% as fair, and 4.3% as poor.

With respect to understanding of the prescribed metabolic diet, 68% rated their understanding as good, 27.7% as fair, and 4.3% as poor. Higher educational level and longer time since diagnosis were associated with a better understanding of dietary management (r = −0.13, *p* = 0.030; r = −0.13, *p* = 0.029).

**Figure 1:**
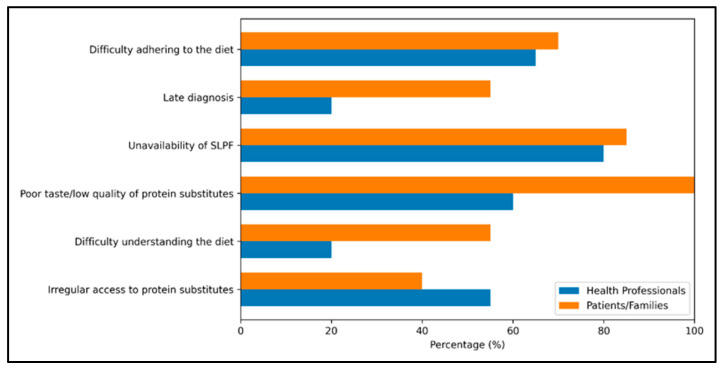
Perceived challenges in dietary treatment. Mean scores (0–100) from professionals and patients/caregivers. SLPF: special low-protein foods.

The majority of respondents (92.8%) reported that the metabolic diet had a higher cost than a regular diet. Regarding screening for risk of food insecurity (*TRIA*), 52.5% of respondents answered affirmatively to question 1 (“In the past three months, did the food run out before you had money to buy more?”), and 43.7% answered affirmatively to question 2 (“In the past three months, did you have to manage with only a few foods to feed household members under 18 years of age because the money ran out?”). Perceptions of the impact of treatment-related barriers from the perspectives of patients and healthcare professionals are presented in Figure 1.

#### 3.2.1. Protein-Restricted Diets

Among patients in regular use of amino acid-based metabolic formula (*n* = 193; 69.4% of the sample), 92% reported fractional intake at least three times per day. Regarding palatability of the formula in use, 44.2% rated it as “very poor”, 35.8% as “poor”, 9.1% as “good”, 8.5% as “neutral”, and 2.5% as “excellent”. With respect to access to metabolic formula, 36% (*n* = 70, including one patient with PKU and the remaining with other IEM) reported problems in the previous year, characterized by insufficient supply relative to the prescribed amount. Among these, 53% reported a lack of formula for periods longer than one month.

Regarding the prescribed amount of protein/amino acids, 74.5% reported knowing their permitted daily intake, whereas 25.5% were unaware of this information. Concerning the use of special low-protein foods, 58.6% reported regular use (three times per week or more). The most common means of acquisition was purchase with personal financial resources (84.8%), followed by donations (8.9%) and provision by the state (6.3%).

#### 3.2.2. Glycogen Storage Diseases

Among the 72 patients with glycogen storage disease (GSD) included in the study, most had GSD type Ia (*n* = 54; 75%), followed by type Ib (*n* = 8; 11%) and type IX (*n* = 8; 11%). All patients were receiving dietary treatment. Most patients (*n* = 54; 75%) performed daily capillary blood glucose monitoring (76% of patients with GSD type Ia and 100% of those with GSD type Ib). Among those performing glycemic monitoring, most used traditional capillary blood glucose meters (*n* = 49; 91%), and 63% of these performed three or more measurements per day.

Regarding the use of uncooked cornstarch, 68 patients (94.4%) reported regular use, whereas 4 (5.5%) did not use it (*n* = 2 with GSD type Ia, *n* = 1 with GSD type V, and *n* = 1 with GSD type IX). Thirty-three patients (45.8%) were on protein supplements: 33/54 with GSD type Ia, 4/8 with GSD type Ib, 7/8 with GSD type IX, 1/1 with GSD type III, and 1/1 with GSD type V.

### 3.3. Treatment Challenges

Healthcare professionals, patients, and family members identified the main perceived challenges related to dietary treatment. These data are presented in Figure 1.

## 4. Discussion

This study provides the first nationwide assessment of the nutritional management of IEM in Brazil, delineating the profile of healthcare professionals, patients, and caregivers, and the challenges families face in accessing optimal treatment.

The participation of professionals from 20 out of the 26 Brazilian states demonstrates relevant national representativeness; however, regional gaps remain, particularly in the North and Midwest regions, which had fewer participants. This finding may reflect the concentration of IEM reference services in the Southeast and South regions—wealthier areas with greater availability of specialized resources and diagnostic capacity [6].

The predominance of dietitians among professionals involved in dietary management reflects the central role of these professionals in the care of individuals with IEM, as dietary therapy is the primary strategy for disease control in many of these conditions. Nevertheless, the fact that most professionals reported having completed only short-term training courses in the field suggests limitations in continuing education and in the consolidation of experienced and highly specialized teams. In Brazil, there are currently no standardized national criteria defining the competencies required for nutritionists working in the management of IEM, nor are there structured specialization programs or formal training pathways designed for practice within newborn screening or rare disease centers. As a result, professional qualification often depends on individual clinical experience and local institutional initiatives, leading to significant heterogeneity in care delivery across regions. This finding underscores the need for structured, long-term training and capacity-building programs that promote technical updating and multidisciplinary integration—key elements for successful treatment outcomes [2,8]. In addition, dietitians were identified as the main source of dietary guidance for families, followed by peer counseling among families. This latter finding highlights the importance of support networks and shared experiences in coping with complex chronic conditions, reinforcing the need to strengthen caregiver support groups and ongoing educational initiatives [8,9].

The substantial number of patients followed by each professional suggests a high demand for care and potential overload of specialized services. This scenario may compromise individualized follow-up and the continuous adjustment of nutritional prescriptions, particularly in regions with limited availability of reference centers [8]. Furthermore, insufficient access to laboratory tests required for medical and nutritional monitoring may negatively affect both short- and long-term follow-up, as well as clinical decision-making [10]. In these situations, therapeutic adjustments are often based primarily on clinical evaluation, anthropometric measurements, dietary records, and symptom assessment, which may compromise accurate metabolic monitoring and individualized nutritional management. Similar findings were reported in a study evaluating PKU management in Latin America, which highlighted significant inequalities in access to diagnostic and dietary resources. Although most countries have newborn screening programs, there remains a shortage of specialized nutritional resources, food composition databases, trained professionals, laboratory support, and accessible therapeutic options [11].

Patients and families reported significant socioeconomic challenges affecting the management of IEM. Many perceived the metabolic diet as more expensive than a regular diet, and a high proportion screened positive for food insecurity, reflecting the vulnerability of these families. In Brazil, according to 2023 PNAD Contínua data, 27.6% of households experienced some level of food insecurity [12]. Importantly, despite the potential underrepresentation of highly vulnerable groups in our survey, substantial barriers related to treatment access and food insecurity were still identified, suggesting that the true magnitude of these challenges may be even greater than that captured in our findings [13,14,15].

These findings suggest that economic constraints may directly affect treatment adherence. Similar barriers have been reported internationally, where the high cost of special foods limits adherence to metabolic diets. Having a reimbursement policy for special foods has been associated with better metabolic control in countries like China [16] and Saudi Arabia [17]. The irregular provision of metabolic formulas reported by many participants may further compromise nutritional status and metabolic control, particularly for non-PKU IEM, which still lack a national policy ensuring formula coverage [17,18,19]. A critical contradiction has been identified even in high income countries such as the U.S.: although newborn screening enables the early identification of these conditions, access to treatment is hindered by high costs and inconsistent insurance coverage [1]. Food insecurity therefore represents a significant barrier to effective dietary treatment, underscoring the need for broader health policies in Brazil that ensure access to the range of special foods required for metabolic diets.

Most participants reported having received their diagnosis five or more years prior to the study, and most PKU cases were diagnosed through newborn screening. This finding reinforces the importance of the Brazilian National Newborn Screening Program (Programa Nacional de Triagem Neonatal—PNTN) as an essential tool for the early diagnosis of several treatable metabolic conditions [20]. The high proportion of diagnoses made after symptom onset (approximately 39%) likely reflects gaps in the national newborn screening program, which does not yet include conditions such as glycogen storage diseases, organic acidemias, and other non-PKU amino acid disorders. Furthermore, significant regional disparities persist; some states in the North and Northeast regions continue to face substantial challenges, with coverage rates as low as 23–68% of live births [14,15,21].

The positive self-perception of both metabolic control and understanding of the prescribed diet suggests that a substantial proportion of the study population feels confident regarding treatment management. However, these findings should be interpreted with caution, as perceptions of metabolic control may not necessarily reflect actual adherence, biochemical control, or objective comprehension of dietary recommendations. In a randomized, double-blind trial, adults with early-treated PKU were unable to reliably distinguish periods of high phenylalanine exposure, despite believing that they could perceive changes in cognition and mood [22]. The association between higher educational level and better understanding of dietary management indicates that socioeducational factors significantly influence families’ ability to interpret and apply nutritional recommendations [23,24]. A similar effect was observed for time since diagnosis, with longer disease experience contributing to a greater mastery of the recommended dietary practices. These findings emphasize the importance of tailoring nutritional education strategies to different educational levels in order to promote equitable access to knowledge [25,26].

Among GSD patients, the high rates of uncooked cornstarch use and blood glucose monitoring observed in this study indicate substantial adherence to key components of GSD management. These findings may reflect adequate access to specialized metabolic care and nutritional guidance among participating families. Nevertheless, the fact that a subset of patients did not perform regular glucose monitoring or did not use cornstarch underscores the persistence of potential barriers to optimal treatment adherence that warrant further investigation in this population [27,28]. Importantly, key dietary therapies for GSD, including uncooked cornstarch and protein supplements, are not covered by the Brazilian Unified Health System (SUS).

This study has methodological limitations, including the use of online questionnaires for data collection, which may have favored participation by individuals with greater Internet access, stronger engagement with support groups, and greater familiarity with the topic, introducing potential selection bias. Although participants from all Brazilian regions were represented, the predominance of respondents from the Southeast and South reflects the unequal distribution of specialized metabolic services in the country and may have limited the capture of challenges faced in historically underserved regions, such as the North and Midwest. This asymmetry limits the generalizability of the findings to the national context.

It is also important to note that IEM comprise a highly heterogeneous group of disorders with diverse dietary requirements, and several subgroups included a small number of participants, limiting more in-depth stratified analyses. The limited number of Brazilian studies focused on the nutritional management of IEM reinforces the relevance of the present survey, while also delimiting its interpretative depth.

## 5. Conclusions

Overall, the findings highlight the need for more robust public policies to ensure continuous and equitable access to metabolic formulas, specialized nutritional support, and health education initiatives targeting both caregivers and healthcare professionals within the primary care network. Beyond regional inequalities, the observed influence of educational level on dietary understanding, together with the high prevalence of food insecurity, underscores that the nutritional management of IEM in Brazil requires an integrated and multidisciplinary approach. This approach should combine specialized clinical care, governmental financial support, structured professional training and education aimed at improving treatment adherence, and reducing health disparities.

## Figures and Tables

**Table 1:** Characteristics of healthcare professionals involved in the dietary management of inborn errors of metabolism (IEM) in Brazil (*n* = 37).

Variable		*n* (%)
Gender	Female	34 (92%)
Occupation	Dietitian	30 (81%)
	Physician	7 (19%)
Theoretical training in IEM	None	13 (35%)
	Short-term course	15 (40%)
	Specialization	6 (16%)
	Master’s degree	5 (13.5%)
	PhD	3 (8.1%)
Years of experience in the field	≤2 years	6 (16.2%)
	3–5 years	8 (21.6%)
	6–10 years	4 (10.8%)
	>10 years	19 (51.3%)
Patients under care	<20	6 (16.2%)
	20–100	18 (48.6%)
	>100	4 (10.8%)
	Not informed	9 (24.3%)
Hours dedicated to IEM	<5 h/week	10 (27%)
	5–10 h/week	8 (21.6%)
	11–20 h/week	7 (18.9%)
	21–30 h/week	6 (16.2%)
	>30 h/week	6 (16.2%)
Type of service	Outpatients only	19 (51.3%)
	Inpatients + outpatients	18 (48.6%)

**Table 2:** Characteristics of the Brazilian sample of patients and caregivers with IEM under dietary treatment (*n* = 278).

Variable		*n* (%)
Female		226 (81.3%)
Respondent’s age (years)	18–24	14 (5%)
	25–34	75 (27%)
	35–44	133 (47.8%)
	45–54	37 (13.3%)
	55–64	10 (3.6%)
	>65	9 (3.2%)
Patient’s current age	0–11 months	16 (5.8%)
	1–4 years	67 (24.1%)
	5–9 years	73 (26.3%)
	10–17 years	64 (23%)
	18–24 years	24 (8.6%)
	25–34 years	26 (9.4%)
	35–44 years	8 (2.9%)
Educational level	Incomplete elementary school	14 (5%)
	Complete elementary school	14 (5%)
	Incomplete high school	21 (7.6%)
	Complete high school or incomplete higher education	106 (38.1%)
	Completed higher education	64 (23%)
	Postgraduate education	59 (21.2%)
Diagnosis	Tyrosinemia	4 (1.4%)
	Organic acidemia *	12 (4.3%)
	Maple syrup urine disease	7 (2.5%)
	Phenylketonuria	168 (60.4%)
	Glycogen storage disease	72 (25.9%)
	Galactosemia	3 (1.1%)
	Classical homocystinuria	9 (3.2%)
	Other **	3 (1.1%)

* Type 1 glutaric aciduria *n* = 3; Propionic acidemia *n* = 4; Methylmalonic acidemia *n* = 3; Others, *n* = 2; ** Others: Hereditary fructose intolerance *n* = 1, Biotinidase deficiency *n* = 1, Fatty acid β-oxidation defect *n* = 1.

## Data Availability

The data presented in this study are available on request from the corresponding author.

## Abbreviations

The following abbreviations are used in this manuscript:

IEM	Inborn Errors of Metabolism
PKU	Phenylketonuria
GSD	Glycogen Storage Disorders
SLPF	Special Low Protein Foods
SUS	Sistema Único de Saúde
